# Mitochondrial Dynamics and Liver Cancer

**DOI:** 10.3390/cancers13112571

**Published:** 2021-05-24

**Authors:** María Isabel Hernández-Alvarez, Antonio Zorzano

**Affiliations:** 1 Departament de Bioquímica i Biomedicina Molecular, Facultat de Biologia, Universitat de Barcelona, 08028 Barcelona, Spain; 2 CIBER de Diabetes y Enfermedades Metabólicas Asociadas, 08028 Barcelona, Spain; 3 Institut de Biomedicina, Universitat de Barcelona, IBUB, 08028 Barcelona, Spain; 4 Institute for Research in Biomedicine (IRB Barcelona), Barcelona Institute of Science and Technology (BIST), 08028 Barcelona, Spain

**Keywords:** hepatocellular carcinoma, chronic liver disease, NASH, liver fibrosis, insulin resistance, Mitofusin 1, Mitofusin 2, OPA1, DRP1

## Abstract

**Simple Summary:**

Hepatocellular carcinoma is a leading cause of cancer-related death worldwide. Major risk factors in liver cancer development include chronic hepatitis B or C virus, autoimmune hepatitis, diabetes mellitus, alcohol abuse, and several metabolic diseases, among others. Standard therapy shows low efficacy, and there is an urgent need for novel therapies. Recent data permit to propose that proteins that control mitochondrial morphology through changes in mitochondrial fusion or mitochondrial fission, confer susceptibility or resistance to the development of liver cancer in mouse models. Here, we review the data that suggest mitochondrial dynamics to be involved in the development of liver tumors.

**Abstract:**

Hepatocellular carcinoma (HCC) is the most prevalent primary liver cancer. Due to its rising incidence and limited therapeutic options, HCC has become a leading cause of cancer-related death worldwide, accounting for 85% of all deaths due to primary liver cancers. Standard therapy for advanced-stage HCC is based on anti-angiogenic drugs such as sorafenib and, more recently, lenvatinib and regorafenib as a second line of treatment. The identification of novel therapeutic strategies is urgently required. Mitochondrial dynamics describes a group of processes that includes the movement of mitochondria along the cytoskeleton, the regulation of mitochondrial morphology and distribution, and connectivity mediated by tethering and fusion/fission events. In recent years, mitochondrial dynamic processes have emerged as key processes in the maintenance of liver mitochondrial homeostasis. In addition, some data are accumulating on the role played by mitochondrial dynamics during cancer development, and specifically on how such dynamics act directly on tumor cells or indirectly on cells responsible for tumor aggression and defense. Here, we review the data that suggest mitochondrial dynamics to be involved in the development of liver tumors.

## 1. Introduction to Liver Cancer

Liver cancer is the fourth leading cause of cancer mortality worldwide, with more than 840,000 new cases and 780,000 deaths currently recorded globally per year [1]. Hepatocellular carcinoma (HCC) accounts for 85% of all primary liver cancers. Early HCC detection can sometimes permit its cure by surgical resection. However, HCC is more often diagnosed as an advanced disease, for which therapy can only slightly extend life expectancy, and, to date, it is under Orphan Drug Designation in Europe and the USA. Standard therapy for advanced-stage HCC is based on anti-angiogenic drugs such as sorafenib and, more recently, lenvatinib [2] and regorafenib as a second line of treatment [3]. However, the resistance and overall survival rates are still unsatisfactory. All three drugs target a variety of cellular tyrosine kinases in addition to vascular endothelial growth factor (VEGF) receptors; sorafenib also targets the cytosolic kinases RAF1 (Raf-1 Proto-Oncogene, Serine/Threonine Kinase also known as CRAF) and BRAF (B-Raf proto-oncogene serine/threonine-protein kinase), and lenvatinib and regorafenib share the receptor tyrosine kinase KIT as a common target. Due to the limited survival benefits of treatment with sorafenib, new therapeutic approaches have been tested. In this regard, given the relevant role of both the RAS/RAF and AKT/mTOR pathway in HCC, several clinical trials have tested the efficacy of sorafenib in combination with mTOR (mechanistic target of rapamycin) inhibitors. The evidence suggests that dual therapy does not improve the efficacy of sorafenib alone [4,5]. Several other studies with multitargeted kinases have concluded that these are not the only signaling networks involved in HCC etiology and/or drug resistance [6].

More recently, immune checkpoint inhibitors have been introduced into HCC clinical practice with high expectations. Since the approval of nivolumab by the FDA in 2017 for the treatment of HCC, combination therapy with anti-angiogenic drugs and immunosuppression checkpoint inhibitors has been tested in clinical studies [7]. However, tumor cells compensate for treatments that compromise proliferation and immune escape via altered apoptosis. Alterations in the normal cellular balance between BAX and BCL2 (pro-apoptotic and anti-apoptotic molecules, respectively) dysregulate apoptosis and promote drug resistance [8]. In light of this connection, it seems reasonable to propose additional treatment with compounds that trigger apoptosis in HCC tumors. 

HCC development and progression are influenced by genetic, epigenetic, and environmental factors. HCC risk factors include chronic hepatitis B virus (HBV) and/or hepatitis C virus (HCV) infection, autoimmune hepatitis, diabetes mellitus, alcohol abuse, and several metabolic diseases [9,10]. Specifically, nonalcoholic steatohepatitis (NASH) is a major risk factor for cirrhosis and HCC [11]. In addition, a number of epidemiological studies have indicated that overweight and obesity increase susceptibility to liver cancer [12]. This risk is higher in men with morbid obesity, and even modest increases in BMI have been linked to greater risk of death by HCC [12]. 

There are two major pathways for the development of HCC. In most cases, HCC develops in patients with chronic liver disease and cirrhosis [13]. A second minor pathway develops through the malignant transformation of hepatic adenomas [14]. 

Patients with chronic liver disease show hepatic inflammation, fibrosis, and aberrant hepatocyte regeneration. These abnormalities can induce cirrhosis and promote a number of genetic and epigenetic events that lead to the formation of dysplastic nodules, which represent preneoplastic lesions [15]. Similarly, hepatocarcinogenesis induced by chemicals in mice is characterized by foci of altered hepatocytes [16,17]. When injected into mice, these cells cause HCC, thus leading to the concept of HCC progenitor cells [18]. Dysplastic cells are thought to undergo alterations that permit the acquisition of proliferative, invasive, and survival advantages before completing the transition to hepatocellular carcinoma. From a genetic perspective, HCC cells accumulate somatic DNA alterations, including mutations in the *TERT* (telomerase reverse transcriptase) promoter (accounting for 60% of cases), *TP53* (tumor protein 53) (30% of cases), *WNT* (Wingless-type MMTV integration site) signaling genes *CTNNB1* (Catenin Beta 1) (30% of cases) and *AXIN1* (10% of cases), and chromatin remodeling genes *ARID1A* (AT-Rich Interaction Domain 1A) (10% of cases) and *ARID2* (AT-Rich Interaction Domain 2) (5% of cases) [13,15]. 

## 2. Mitochondrial Dynamics in the Liver

Mitochondrial dynamic processes are key to the maintenance of mitochondrial homeostasis [19,20]. Catalyzed by a range of proteins, mitochondrial dynamics include the movement of mitochondria along the cytoskeleton, the regulation of mitochondrial architecture, and connectivity mediated by tethering and fusion/fission events [19,20,21]. Mitochondrial dynamics is a relevant aspect of mitochondrial biology and function, controlling mitochondrial respiration, mitochondrial quality control, autophagy, apoptosis, and Ca^2+^ homeostasis. As a result of this, changes in mitochondrial dynamics impact cellular function. Indeed, alterations in this process have been associated with neuropathies (Charcot–Marie–Tooth type 2A and autosomal dominant hereditary optic neuropathy or ADOA) [22,23,24], neurodegenerative disorders such as Parkinson’s disease [25], atherosclerosis [26], and metabolic diseases such as obesity and type 2 diabetes [27,28,29]. 

**Figure 1:**
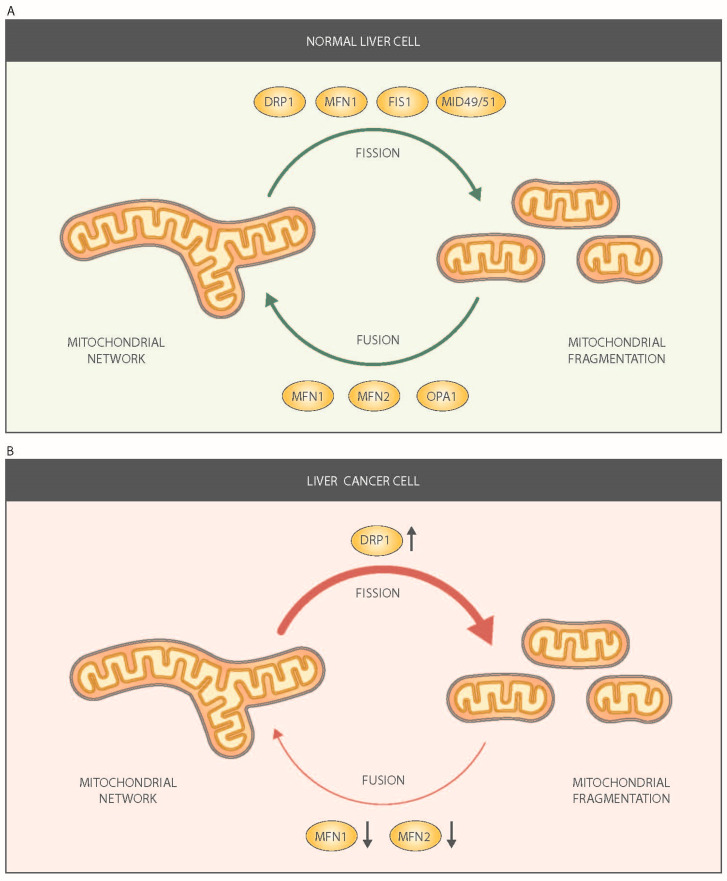
Mitochondrial dynamics in normal (**A**) and tumor liver cells (**B**). Mitochondrial fusion is a two-step process in which outer mitochondrial membrane (OMM) fusion is mediated by homo-and hetero-oligomeric complexes between mitofusin1 (MFN1) and mitofusin2 (MFN2), and inner mitochondrial membrane (IMM) fusion is mediated by optic atrophy gene 1 (OPA1). Mitochondrial fission is mediated by the action of dynamin-related protein 1(DRP1), which is recruited to mitochondria by different receptors: fission 1 homolog protein (FIS1), mitochondrial fission factor (MFF), mitochondrial dynamics protein of 49 kDa (MID49), and mitochondrial dynamics protein of 51 kDa/mitochondrial elongation factor 1 (MID51/MIEF1).

Mitochondrial dynamics encompasses mitochondrial fusion and fission events (Figure 1A) [30]. Mitochondrial fusion in mammals is catalyzed by the proteins Mitofusin 1/MFN1, Mitofusin 2/MFN2, and optic atrophy gene 1/OPA1. They are dynamin-related proteins with GTPase activity. The two mitofusins are located in the outer mitochondrial membrane, whereas OPA1 is located in the inner mitochondrial membrane and in the intermembrane space (Figure 1A). Mitochondrial fusion proteins show ubiquitous expression. Nevertheless, MFN1 is highly expressed in heart, liver, pancreas, adrenal glands, and testis, whereas MFN2 is more abundant in heart, skeletal muscle, brain, and brown adipose tissue [27,31]. OPA1 is highly expressed in brain, retina, liver, testis, heart, and skeletal muscle [23,24]. The complexity of the biology of OPA1 is high due to the fact alternative splicing generates eight distinct OPA1 isoforms in humans, and they show different functional activities in mitochondria [32]. 

**Figure 2:**
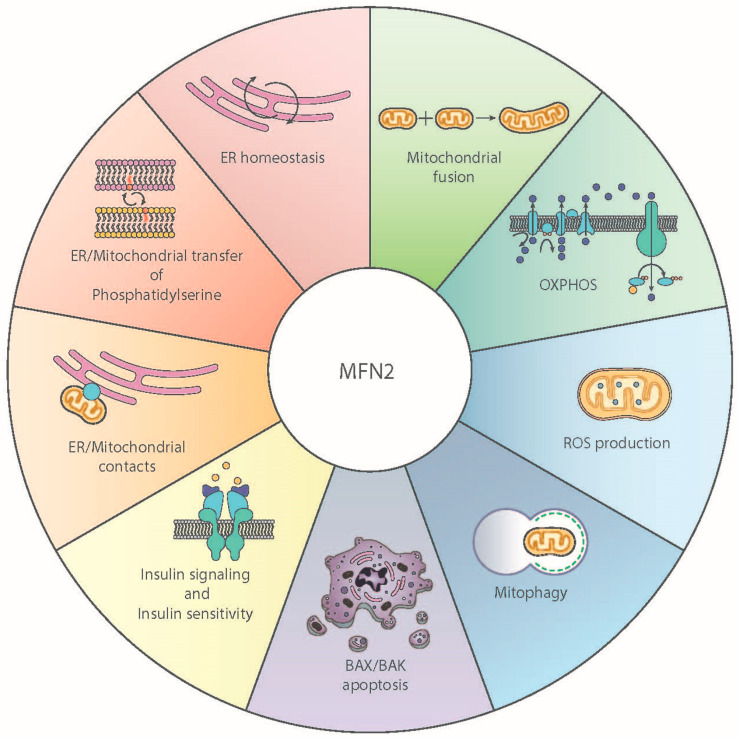
Functional role of Mitofusin 2. Schematic overview of the multiple functional roles played by Mitofusin 2 (MFN2) in mammalian cells.

In addition to its role as a mitochondrial fusion protein, MFN2 also serves as an endoplasmic reticulum (ER) protein in the connection of ER membranes to mitochondria (Figure 2) [33], and its depletion causes ER stress and plays a relevant role in the maintenance of mitochondrial metabolism, insulin signaling, and energy homeostasis [27,28,34,35,36]. MFN2 has recently been reported to participate in the transfer of phosphatidylserine from ER to mitochondria, which seems to be a central activity in many of the molecular and cellular functions of the protein [36].

Mitochondrial fission is catalyzed by DRP1 (Dynamin-related protein 1), MID49, MID51, FIS1 (Fission 1 homolog protein), and MFF (mitochondrial fission factor) (Figure 1A). DRP1 has GTPase activity. It is located in the cytosol and shows a broad tissue distribution [37]. Mitochondrial fission requires the recruitment of DRP1 to the outer mitochondrial membrane, where it forms a ring-like structure located on the future mitochondrial scission site [38]. In order to localize on the mitochondrial membrane, DRP1 must interact with FIS1 [39] and MFF [40], both integral proteins of the outer mitochondrial membrane. In contrast to other fusion or fission proteins, FIS1 and MFF do not show GTPase activity. Recently, mitochondrial dynamic proteins of 49 and 51 kDa (MID49 and MID51, respectively) have been described to participate in the recruitment of DRP1 to mitochondria, although their role in mitochondrial fission is not well established [41,42,43]. 

The physiological relevance of mitochondrial dynamics has been studied in mammalian tissues upon genetic manipulation of specific genes. Ablation of mitochondrial fusion and fission proteins in mice is embryonically lethal [44,45,46,47,48], thus highlighting the importance of mitochondrial dynamics in physiology. Studies in conditional KO mice and genetic manipulation have revealed some of the roles of these proteins in liver cell physiology. Ablation of MFN2 in mouse hepatocytes alters mitochondrial morphology and reduces mitochondrial respiratory complexes I and II [28]. MFN2 ablation in mouse liver also causes deficient transfer of phosphatidylserine from the ER to mitochondria. This dysregulation has two major functional implications: (a) it reduces the synthesis of phosphatidylethanolamine (PE) in mitochondria, and (b) it leads to ER stress, inflammation, and fibrosis [35,36]. Hepatic MFN2 is also essential for normal insulin signaling and glucose homeostasis [28]. In contrast to MFN2, hepatic MFN1 deficiency increases mitochondrial content and also causes enhanced mitochondrial respiration through complexes I and II [49]. Under these conditions, mice are protected against high-fat-diet-induced insulin resistance and ER stress is not induced [49]. Available data allow us to propose that hepatic ablation of MFN2 or MFN1 induces distinct stress signals, thereby leading to opposite patterns of metabolic alterations.

Loss of hepatic OPA1 also alters the morphology of mitochondria [50]. Simultaneous loss of DRP1 and OPA1 normalizes mitochondrial size in hepatocytes, and OPA1 ablation rescues liver damage induced by a methionine-choline-deficient diet [50]. In contrast, mitochondrial size in DRP1-knockout hepatocytes increases, and mitochondria are elongated [50]. Hepatic ablation of DRP1 mice also protects against a high-fat diet in the presence of ER stress, and enhances hepatic expression of FGF21 [51]. 

The expression of the mitochondrial fusion protein MFN2 is subject to regulation in the liver via hormonal and metabolic factors. Glucocorticoids repress MFN2 expression in hepatoma cells and in mouse liver [52]. The pro-inflammatory factor TNFα also reduces MFN2 expression in human embryo liver cells [53] and in response to liver ischemia-reperfusion injury [53]. In addition, mice subjected to a high-fat diet show reduced hepatic MFN2 in parallel to insulin resistance and oxidative stress [54]. Induction of lipid accumulation in hepatoma HepG2 cells by incubation with long-chain fatty acids represses MFN2, whereas omega-3 polyunsaturated fatty acids such as eicosapentaenoic acid or docosahexaenoic acid increase MFN2 [55]. Furthermore, patients with extrahepatic cholestasis show low hepatic MFN2 [56], and glycochenodeoxycholic acid, the main toxic component of bile acid in these patients, represses MFN2 in human liver cells [56].

## 3. Mitochondrial Dynamics in Tumor Cells

There are some data on the role of mitochondrial dynamics in tumor cells and in connection with cells responsible for tumor aggression and/or defense [57,58]. Tumor cells are characterized by a series of defined steps that ultimately lead to malignant transformation. The most common steps are resistance to cell death, deregulated cell metabolism and constant reactive oxygen species (ROS) production, cell hyperproliferation, increased invasive power, uncontrolled mitophagy, and finally tissue vascularization. It is noteworthy that mitochondrial dynamics are involved in all these processes, both under physiological conditions and during neoplastic transformation [58,59]. Different types of cancer (and even different stages of the same cancer) are known to modulate the balance between mitochondrial fusion and fission. Several studies have reported that the expression of mitochondrial dynamic proteins such as DRP1, MFN1, and MFN2 is dysregulated in lung, bladder, and breast cancers in humans [60,61,62]. It has been also reported that reduced MFN2 levels in breast tumors are associated with poorer outcomes, and cell lines with silenced MFN2 display increased viability and aggressiveness, which seems to be mediated by mTORC2/AKT [63]. In bladder cancer, reduced MFN2 expression levels, possibly mediated by the WNT/β-catenin pathway, also correlate with poorer prognosis [64]. Therefore, dysregulation of mitochondrial dynamics is closely involved with tumorigenesis and tumor progression [58,59]. In addition, mitochondrial dynamics may influence cancer cell resistance, although the mechanisms are unclear [3]. It has been proposed that mitochondrial fusion promotes tumor cell resistance to apoptosis, whereas mitochondrial fission has been associated with increased invasiveness and proliferation.

## 4. Mitochondrial Fusion Proteins and Liver Cancer

There are some data indicating the presence of alterations in mitochondrial fusion proteins in HCC. Some results have revealed a lower average mitochondrial length in HCC tissues compared to that in adjacent nontumor tissues [65]. These observations point to an altered balance between mitochondrial fusion and fission in HCC tumor cells (Figure 1B). In this regard, reduced expression levels of MFN1 protein and mRNA have been reported in HCC tissue compared to adjacent nontumor tissue [65,66]. In addition, low MFN1 protein levels in human HCC correlate with vascular invasion and poor prognosis [65,66], and the expression of MFN1 is reduced in distant metastases of HCC when compared to primary HCC [67]. Thus, a decrease in MFN1 is a main candidate associated with HCC metastasis [66]. Interestingly, MFN1 depletion triggers the epithelial-to-mesenchymal transition of HCC [65], and MFN1-deficient HCC cells show lower E-cadherin values and increased mesenchymal markers. These results have been confirmed by subcutaneous xenographs in mouse models and they further support the notion that MFN1 strongly decreases the metastatic potential of HCC cells. In this context, it has been proposed that MFN1 regulates metastasis in HCC by switching cellular metabolism from glycolysis to oxidative phosphorylation, and treatment with the glycolysis inhibitor 2-deoxyglucose suppresses the effects induced by MFN1 deficiency [66].

HCC tumors are frequently characterized by loss of heterozygosity in the MFN2 gene [68]. In some studies, HCC tissue showed reduced MFN2 protein and mRNA expression compared to nontumor tissue [69,70,71], whereas other studies reported no differences [65]. In addition, a negative correlation between MFN2 levels and the prognosis of liver cancer has been described [69,71,72]. Thus, Kaplan–Meier survival curves and multivariate analyses show that low MFN2 expression in HCC indicates poor prognosis and is linked to worse survival [69,71,72]. Interestingly, tumor MFN2 mRNA expression was found to significantly correlate with gender and preoperative alpha-fetoprotein levels [69]. Therefore, it is possible that hepatic MFN2 is differentially regulated in men and women. Concerning this, miR-761 microRNA may regulate MFN2 in HCC. Thus, miR-761 is upregulated in HCC, and an inhibitor of miR-761 upregulates MFN2, which suppresses tumor growth and metastasis both in vivo and in vitro [73].

It has also been reported that MFN2 overexpression in HCC cells reduces cell proliferation and induces spontaneous apoptosis [70]. This apoptosis was characterized by reduced mitochondrial membrane potential (ΔΨm), lower concentrations of calcium Ca^2+^ ions in the ER, and high concentrations of reactive oxygen species (ROS) and mitochondrial Ca^2+^ [71]. In addition, MFN2 overexpression reduces cell cycle arrest in S phase, increases caspase-3 activation and PARP cleavage, recruits BAX to mitochondria, and decreases cytochrome c in mitochondria [61]. In turn, BAX and BAK participate in MFN2-dependent mitochondrial morphology [74]. MFN2 seems to promote apoptosis and inhibit proliferation in HCC cells through BAX/BCL-2 [70]. Nevertheless, it is unclear whether the reported effects of MFN2 overexpression show a degree of nonspecificity. 

Consistent with the concept that MFN2 plays an important role in the biology of the liver, hepatic MFN2 depletion promotes the generation of liver tumors in mice both with aging and in response to carcinogens [36]. 

It has also been proposed that mitochondrial impairment contributes to the pathogenesis of chronic liver diseases. Endogenous MFN2 expression has been reported to decrease in patients with extrahepatic cholestasis characterized by elevated levels of toxic bile acids [56], and also in subjects with NASH [36]. Moreover, hepatic depletion of MFN2 in mice causes a NASH-like condition characterized by hepatic steatosis, inflammation, and fibrosis [36]. Regarding liver fibrosis, which is a risk factor in the development of cirrhosis and HCC [75], high expression of MFN2 protein inhibits the Transforming growth factor beta 1/Smad (TGF-β1/Smad) signaling pathway, thus triggering the downregulation of type I, type III, and type IV collagen and reducing the formation of factors associated with liver fibrosis [76]. Furthermore, MFN2 overexpression using AAV-MFN2 was found to improve carbon-tetrachloride-induced liver fibrosis in vivo [76]. In keeping with these observations, specific deletion of MFN2 in liver causes ER stress, which leads to a chronic inflammatory state that in turn causes the activation of TGF-β1 and deposition of collagen, with the subsequent development of liver cancer [36].

OPA1 is a major mitochondrial fusion protein, and it also regulates crista formation. Some data indicate that HCC tumors do not show changes in OPA1 protein or mRNA expression compared to adjacent tissue [65,77]. It is likely that OPA1 expression is maintained in HCC tumors in order to preserve crista formation. In this regard, it would be useful to document whether the expression of all OPA1 isoforms is high under those conditions. Along these lines, OPA1 participates in apoptosis by regulating the rate and extent of cytochrome c during apoptosis. The effects of OPA1 on apoptosis depend on its GTPase activity, its oligomerization, and its processing to soluble forms by the rhomboid protease PARL [78,79]. 

**Figure 3:**
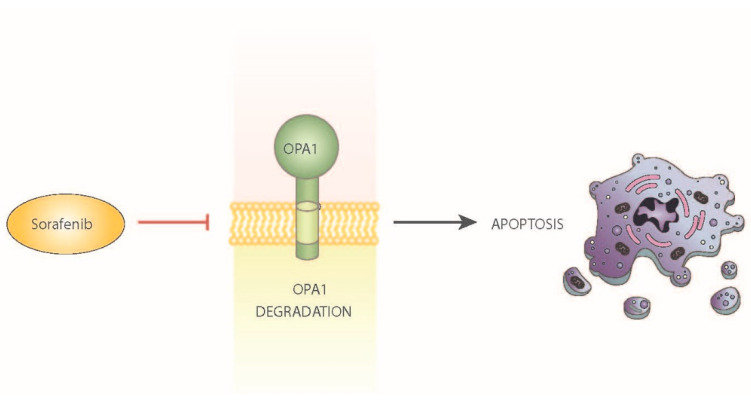
Effect of sorafenib on mitochondrial dynamics. Schematic overview of the effects of sorafenib, a first-line therapy in HCC, as an inhibitor of OPA1 and activator of apoptosis.

Interestingly, some of the cell death effects of the multikinase inhibitor sorafenib are dependent on the dysregulation of OPA1 [80]. Sorafenib has been reported to induce mitochondrial fragmentation and trigger the release of cytochrome c. Furthermore, within a few minutes, sorafenib reduces OPA1 expression. This observation thus suggests that sorafenib promotes the rapid degradation of OPA1 in mitochondria, thus leading to the activation of apoptosis (Figure 3). It has also been shown that HCC cells can be sensitized to sorafenib-induced apoptosis by prior depletion of OPA1 [80].

In all, available data suggest the existence of alterations in the expression of MFN1 and MFN2 in HCC cancer cells, and that these alterations could be instrumental in the biological properties of those cells. In contrast, the expression of OPA1 is maintained at a high level in HCC cells, which may be key in prevention of apoptosis. Further studies are required to thoroughly analyze the roles of these proteins in the development and progression of HCC. These data also raise the possibility of using the mitochondrial fusion proteins as drug targets for the treatment of HCC.

## 5. Mitochondrial Fission Proteins and Liver Cancer

Many studies have reported that increased mitochondrial fission is possibly proto-tumorigenic and is associated with oncogene expression, metabolism, cellular behavior, and responses to stress [81,82]. It has been proposed that mitochondrial fission is activated in HCC tumors based on the shorter mitochondrial size and on the enhanced expression of DRP1 protein and mRNA levels [65,66,83]. Analysis of the activation state of DRP1 in HCC tumors is lacking, so further studies are warranted. The expression of the putative mitochondrial DRP1 receptor FIS1 is not altered in HCC tumors [65].

The enhanced expression of DRP1 in HCC cells is of potential biological relevance. It has been reported that dexamethasone upregulates the expression DRP1 in hepatoma FaO cells. Moreover, the effects of dexamethasone on mitochondrial respiration and on gluconeogenic activity depend on normal DRP1 activity. In this regard, inactivation of DRP1 activity via expression of a dominant-negative form of DRP1 impairs the effects of dexamethasone on oxygen consumption and on mitochondrion-dependent glucose production from lactate/pyruvate [52]. 

Importantly, overexpression of DRP1 in HCC cells has been linked to enhanced tumor growth under in vivo conditions, and, vice versa, DRP1 deficiency caused a reduced tumor growth [65,84]. DRP1-mediated mitochondrial fission promotes cell proliferation through the crosstalk of the p53 and NF-κB pathways in hepatocellular carcinoma [85]. Moreover, HCC cell survival mediated by enhanced mitochondrial fission seems to be dependent on facilitation of autophagy and inhibition of mitochondrion-dependent apoptosis [65]. It has also been reported that the survival-promoting role of increased mitochondrial fission is mediated by elevated ROS production and subsequent activation of AKT, which facilitates mouse double minute 2 homolog (MDM2)-mediated degradation of TP53 and NFKappaΒ Inhibitor Alpha (NFKBIA) and the nuclear factor-κB (IκB) kinase (IKK)-mediated transcriptional activity of NFKappaB in HCC cells [65]. 

Interestingly enough, it has been reported that the expression of DRP1 is strongly increased in distant metastases of HCC compared to in primary HCC [67]. In addition, DRP1-overexpressing tumor cells caused a greater incidence of intrahepatic and lung metastasis in an orthotopic nude mouse model [67]. In all, these data support the view that the mitochondrial fission protein DRP1 plays a role in the regulation of cell migration and metastasis.

It is likely that a number of proteins modulate DRP1 in tumor cells. The role of the large tumor suppressor 2 (LATS2), a mediator of the cell apoptotic response pathway, was proposed in a previous study [86]. LATS2 overexpression in HCC cells induces DRP1 and promotes mitochondrial fragmentation [86]. Further studies are required to provide detailed mechanistic information about this potential pathway.

In all, the available information seems to indicate that increased mitochondrial fission plays a critical role in regulating HCC cell survival, providing strong evidence for this process as a drug target in the treatment of HCC.

## 6. Future Perspectives

In recent years, there have been interesting advances in the study of mitochondrial dynamics in connection with cancer. Specifically, HCC, the most common type of liver cancer, has been clearly linked to a dysregulation of mitochondrial dynamics. The majority of the available data support the proposal that mitochondrial dynamic processes are influenced in HCC tumors, in that mitochondrial fusion is reduced whereas mitochondrial fission is more active than in nontumor tissue. This proposal requires thorough demonstration as well as resolution of the mechanisms that operate in HCC tumors.

Available data also suggest that mitochondrial fragmentation of HCC tumors plays a key role in cell proliferation and migration as documented in vitro and in liver tumor growth and metastasis studied using nude mice. Based on these observations, it seems relevant to explore whether mitochondrial dynamics could present pharmacological targets for the treatment of HCC, and perhaps other types of cancer. 

A fundamental question raised by the available evidence is whether the alterations reported in mitochondrial dynamic proteins in HCC tumors are also present in the initial phases of tumor promotion and growth. In regard to this connection, it will be key to analyze mitochondrial dynamics in HCC progenitors, and to analyze whether enhanced mitochondrial fission or reduced mitochondrial fusion indeed facilitate the tumor initiation, growth, or metastasis of liver cancer cells. 

Clarification of some of the above questions will be relevant not just from the point of view of the fundamental understanding of mitochondrial biology, but also from the translational perspective, and they may permit the identification of novel therapeutic strategies for HCC.

## 7. Conclusions

Currently available data suggest that mitochondrial dynamics plays a relevant role during cancer development, and more specifically in liver cancer. Further studies on the mechanisms by which specific proteins involved in mitochondrial dynamics participate in tumor growth are of great importance. This will lead to the identification of relevant pathophysiological mechanisms and to the discovery of novel drug targets.
